# Comparative transcriptomic analyses of chlorogenic acid and luteolosides biosynthesis pathways at different flowering stages of diploid and tetraploid *Lonicera japonica*

**DOI:** 10.7717/peerj.8690

**Published:** 2020-03-06

**Authors:** Hongli Wang, Yanqun Li, Sibo Wang, Dexin Kong, Sunil Kumar Sahu, Mei Bai, Haoyuan Li, Linzhou Li, Yan Xu, Hongping Liang, Huan Liu, Hong Wu

**Affiliations:** 1 BGI-Shenzhen, Shenzhen, Guangdong, China; 2 BGI Education Center, University of Chinese Academy of Sciences, Beijing, China; 3 State Key Laboratory for Conservation and Utilization of Subtropical Agro-Bioresources, South China Agricultural University, Guangzhou, Guangdong, China; 4 Department of Biology, University of Copenhagen, Copenhagen, Denmark; 5 State Key Laboratory of Agricultural Genomics, BGI-Shenzhen, Shenzhen, Guangdong, China; 6 China National GeneBank, BGI-Shenzhen, Shenzhen, Guangdong, China

**Keywords:** Lonicera japonica, De novo transcriptome assembly, Chlorogenic acid, Luteolosides, Traditional Chinese medicine, Flower development

## Abstract

The Flos Lonicerae Japonicae (FLJ), *Lonicera japonica* Thunb, belonging to the Caprifoliaceae family, is an economically important plant that is highly utilized in traditional Chinese medicine as well as in Japanese medicine. The flowers of these plants are rich in chlorogenic acid (CGA) and luteoloside. Our previous study revealed that tetraploid *L. japonica* has higher fresh/dry weight, phenolic acids and flavonoids contents than those of diploid plants. However, why tetraploid *L. japonica* can yield higher CGA and luteolosides than that in diploid and what is the difference in the molecular regulatory mechanism of these pathways between diploid and tetraploids remained unclear. Therefore, in the present study, we performed comprehensive transcriptome analyses of different flowering stages of diploid and tetraploid *L. japonica*. The CGA content of tetraploid was found higher than that of diploid at all the growth stages. While the luteoloside content of diploid was higher than that of tetraploid at S4 and S6 growth stages. We obtained a high-quality transcriptome assembly (N50 = 2,055 bp; Average length = 1,331 bp) compared to earlier studies. Differential expression analysis revealed that several important genes involving in plant hormone signal transduction, carbon metabolism, starch/sucrose metabolism and plant-pathogen interaction were upregulated in tetraploid compared with the diploid *L. japonica*, reflecting the higher adaptability and resistance of tetraploid species. Furthermore, by associating the phenotypic data and gene expression profiles, we were able to characterize the potential molecular regulatory mechanism of important biosynthetic pathways at different flowering stages. Overall, our work provides a foundation for further research on these important secondary metabolite pathways and their implications in traditional Chinese/Japanese medicine.

## Introduction

*Lonicera japonica* Thunb, belonging to the Caprifoliaceae family, is a stolon shrub featured with perennial, evergreen and twining vine, which has been used as an important raw material in Chinese traditional medicine (TCM) as a treatment various diseases such as to clean away the heat-evil or heal the swelling for over thousands of years (Shang et al., 2011; Rai et al., 2017). The flowers and floral buds are the most important medicinal tissues that have been recorded in the classical pharmacopeia of TCM “Ming Yi Bie Lu” and “Shen Nong Ben Cao Jing” (Rai et al., 2017). *L. japonica* is also known as Flos Lonicerae Japonicae (FLJ) since the young flower is white primitively and it gradually fades to yellow later with its maturity (Rai et al., 2017). *L. japonica* possesses highly commercial value since it is widely used in healthy food, healthy beverages such as “Jin Yin Hua” tea and wine production (Wang, Clifford & Sharp, 2008; Yuan et al., 2014). Modern pharmacological studies suggested that the pharmacological effects of *L. japonica* are diverse and a wide range of bioactive properties, including anti-bacterial, anti-inflammatory, anti-oxidant, anti-viral, anti-angiogenic, antipyretic, hepatoprotective and anti-tumor effects (Lee et al., 2010; Shang et al., 2011; Kong et al., 2017a). Recent studies indicated these functions are mainly attributed to the complex and diverse chemical composition of *L. japonica* (Li, Kong & Wu, 2018). Most recently, Wang et al. (2019) provided a comprehensive characterization of the expression profiles of transcription factors (TFs) during the developmental stages of *L. japonica*.

The main chemical constituents of *L. japonica* extracts include phenolic acids, flavonoids, volatile oils and saponins (Rai et al., 2017). Phenolics and flavonoids have high anti-oxidant activities and pharmacological studies have proven their important role in removing harmful free radicals and in the prevention of diseases, such as inflammation, cardiovascular disease, rheumatoid arthritis and neurodegenerative disease (Kong et al., 2017a; Huyut, Beydemir & Gülçin, 2017). Chlorogenic acid (CGA) and luteolosides are the leading secondary metabolites in *L. japonica*; besides CGA and luteolosides, secoiridoids such as loganin, secologanin, sweroside and kingiside have also been identified from the extracts of *L. japonica* (Rai et al., 2017). CGA is a primary phenylpropanoid generated from the shikimic acid pathways with high anti-oxidant activities. Therefore, it is often used in the form of medicines (Rai et al., 2017). Luteolin, its sugar conjugated derivative lutein, was also derived from the phenylpropanoid metabolic pathway and it is also the major component of *L. japonica* extracts (Lee et al., 2010; Rai et al., 2017). Studies have shown that luteolin, lutein and luteolosides have antioxidant, anti-inflammatory, anti-tumor and anti-5-lipoxygenase activity (Chen et al., 2007). Recent studies have shown that these biological activities of *L. japonica* were mainly attributed to the CGA and luteolosides (Kong et al., 2017a). Therefore, they are now treated as the standard compounds to assess the quality of *L. japonica*.

The polyploidization commonly results in the genome duplication and increasing gene number (Comai, 2005; Soltis et al., 2015). Many previous studies have shown that this process brings significant changes in morphology and physiology and also enhances the growth rates and the level of secondary metabolites. De Weathers & Jesus-Gonzalez (2003) showed tetraploid *Artemisia annua* hairy roots produce more artemisinin than diploids. The tetraploid clone of Egyptian henbane could produce more scopolamine than the diploid counterpart under similar growth conditions (Dehghan et al., 2012). Tetraploid *Matricaria chamomilla* displayed higher productivity of phenolic glucosides than the diploid plants (Repčák & Krausová, 2009). Therefore, polyploid plants usually exhibit increased biological activity than diploid plants. Tetraploid *L. japonica* also has excellent characteristics (e.g., high yield, excellent quality, strong resistance) compared to its diploid plant (Kong et al., 2017a; Li, Kong & Wu, 2018). Our previous study provided the key information that tetraploid *L. japonica* has higher fresh weight, dry weight and phenolic acids and flavonoids contents than those of diploid plants (Kong et al., 2017a). However, why tetraploid *L. japonica* can yield high CGA and luteolosides and what is the difference of molecular regulatory mechanism of CGA and luteolosides biosynthesis pathways between diploid and tetraploid *L. japonica* is also still unclear. In the present study, we performed deep RNA sequencing and gene expression analysis for diploid and tetraploid *L. japonica*. Homologs for all enzymes from CGA, luteolin and other metabolic pathways were identified. We compared the differentially expressed genes (DEGs) at different growth stages between diploid and tetraploid *L. japonica*, associating the gene expression analysis with the phenotypic results to study the molecular regulatory mechanism of CGA and luteolosides biosynthesis pathways between diploid and tetraploid *L. japonica*.

## Materials and Methods

### Plant materials, sample collection and RNA extraction

Fresh buds and flowers were collected from 3-year-old diploid and tetraploid *L. japonica* plants cultured in the medicinal garden of South China Agricultural University in Guangzhou, Guangdong province, China, from 25 March 2015 to 20 April 2015 as described in our previous article (Kong et al., 2017a). Briefly, the juvenile upper branches of the plants were selected for the collection of fresh buds or flowers at six different growth stages, including young alabastrum (S1), green alabastrum (S2), slightly white alabastrum (S3), whole white alabastrum (S4), silvery flower (S5) and golden flower (S6). Plants were identified and validated by Prof. Yue-Shen Yang of South China Agricultural University. Samples were pretreated by collecting individual samples (buds or flowers) from healthy plants of *L. japonica* at different growth stages. Total RNA was extracted from flower samples by using the Concert Plant RNA Reagent (Cat. 12322–012; Invitrogen, Carlsbad, CA, USA) according to the manufacturer’s protocol. The purity, concentration and integrity of RNA samples were measured by Nanodrop, Qubit 2.0 and Agilent 2100 methods (Simbolo et al., 2013), respectively, to ensure the use of qualified samples for transcriptome sequencing.

### High-performance liquid chromatography determination

Samples were pretreated by collecting individual samples (buds or flowers) from healthy plants of *L. japonica* at different growth stages. The desiccation of the sample was performed according to the method Kong et al. (2017a). An accurately weighed powder sample (0.10 g) was suspended in 25 ml 50% methanol v/v, ultrasonically extracted for 40 min and then cooled to room temperature; 50% methanol was added to compensate for lost weight. Each sample was ultrasonically extracted three times and the methanol solution was filtered through a 0.22 µm membrane before HPLC analysis. HPLC determination and analysis was performed according to the methods of Kong et al. (2017a).

### Library preparation, Illumina sequencing and pre-processing of raw reads

RNA samples with RNA integrity number (RIN) value over eight was selected for mRNA preparation, fragmentation, cDNA synthesis and library preparation. The mRNA was broken into short fragments as a template. The first strand of cDNA was then linked to the buffer, RNase H, dNTPs and DNA Polymerase I made into the second chain synthesis reaction system. cDNA was purified using a QiaQuick PCR kit. The samples were purified and the cDNA ends were repaired. The repaired cDNA 3′ end was added with the base “A” and connected to the joint. The size of the fragment was then selected. Finally, PCR was performed to construct the sequencing library. The established library was sequenced by the Illumina HiSeq™ 4,000 platform. The raw data were pre-processed and filtered to exclude the low-quality sequence by using the SOAP filter software.

### De novo transcriptome assembly and unigenes

Trinity software was used to de novo transcriptome assembly in this study and then TGICL software was used to remove the reluctances. The overlapped reads were assembled to contigs. The contigs were joined into scaffolds and the scaffolds were further assembled to unigenes by clustering and removing redundancy. We used the *k*-mer size of 25 (default size) to set the de novo transcriptome assembly. The detailed pipeline is shown in Fig. S1.

### Functional annotation, GO and KEGG classification and analysis of metabolic pathway

BLAST software was used to compare the unigenes sequences with the databases of NCBI non-redundant protein (Nr), Swiss-Prot, Gene Ontology (GO), Clusters of Orthologous Groups (COG), euKaryotic Orthologous Groups (KOG), eggNOG and Kyoto Encyclopedia of Genes and Genomes (KEGG) to obtain the gene functional annotation information. Additionally, HMMER software was used to compare our unigenes with the Protein family (Pfam) database to obtain the domain annotation information of unigenes. In this study, the BLAST parameter *e*-value <10-5 and HMMER parameter *e*-value <10-10 were considered as the threshold. For the GO function classification, unigenes were further analyzed and classified using GO based on the Nr results. Finally, unigenes were classified into three main independent GO categories: molecular function, biological process and cellular component. For the KEGG function classification, unigenes were annotated into five categories of KEGG metabolic pathways: Cellular processes, Environmental information processing, Organismal systems, Metabolism and Genetic information processing. Additionally, the five main categories were divided into 20 small classes.

### Transcriptome expression and DGE profiling analysis

Fragments Per Kilobase of transcript per Million mapped reads (FPKM) was calculated to estimate the expression level of unigenes. In this study, the reads obtained by sequencing were compared with the unigene library by Bowtie and then the expression level was estimated by combining RSEM. FPKM value was used to represent the expression abundance of the corresponding unigene. Finally, the DEseq package was used to select the unigenes with differential expression levels with the parameter of FDR ≤ 0.01 and at least a 2-fold expression change.

### SNPs and SSRs detection

The reads of each sample were compared with the unigenes by STAR and the SNP sites were identified by GATK’s SNP calling process for RNA-seq. There were two main criteria for SNPs identification: First, no more than three single-base mismatches occur continuously within the range of 35 bp, and second, the sequencing read depth coverage for the standardized SNP quality value should be > 2X. According to the above conditions, the SNP site’s information of each sample was finally obtained.

### Statistical analysis

The data are presented as the mean ± SE and there were at least three replicates. The difference of genes expression between diploid and tetraploid plants at different growth stages was analyzed by one-way analysis of variance (ANOVA). A *p*-value of less than 0.05 was considered statistically significant.

## Results

### Chlorogenic acid and Luteoloside level at various growth stages between diploid and tetraploid *L. japonica*

**Figure 1:**
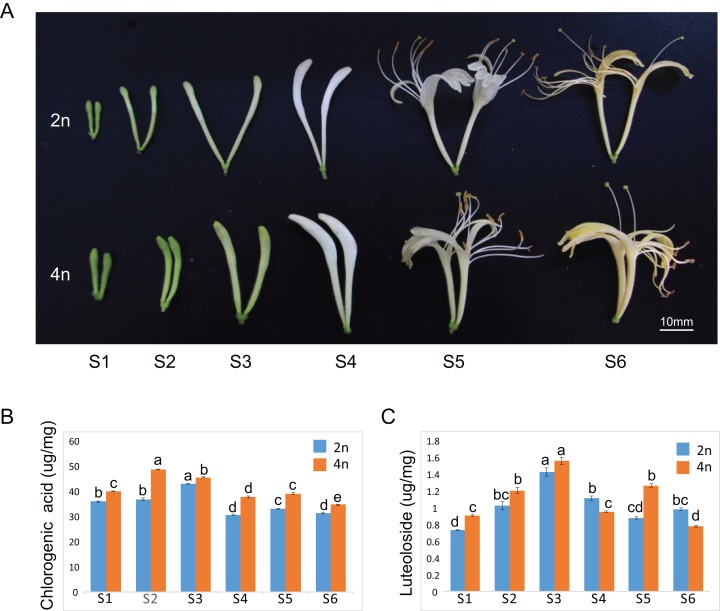
Plant tissues of *L. japonica* used for de novo transcriptome assembly. (A) Six growth stages of diploid (2n) and tetraploid (4n) *L. japonica* buds and flowers were used for the chemical and transcriptome analysis. (B) The changes of chlorogenic acid contents at six growth stages of diploid and tetraploid *L. japonica* buds and flowers in unit weight. (C) The changes of luteolosides contents at six growth stages of diploid and tetraploid *L. japonica* buds and flowers in unit weight. Letters a–e show statistical differences. S1: young alabastrum, S2: green alabastrum, S3: slightly white alabastrum; S4: whole white alabastrum; S5: silvery flower; S6: golden flower. Error bars are ± SE, *n* ≥ 3.

The floral organ diameter of the tetraploid plants was wider than that in the diploid plants at corresponding growth stages. Typical phenotypes of a floral organ are shown in Fig. 1A. The fresh and dry weights of 100 buds or flowers in both diploid and tetraploid plants at six different growths were reported in our previous study, indicating a higher fresh and dry weight of buds by tetraploids (Kong et al., 2017a). A similar observation was made for each growth stage of the tetraploid plants in the present study as well. Our previous study showed remarkable enhancements of CGA and luteoloside yield in the dry samples of 100 buds or flowers (the content × the average biomass per 100 buds or flowers). To further investigate the molecular regulatory mechanism to correlate with gene expression profile, we also measured the CGA and luteoloside contents in a unit weight of diploid and tetraploid *L. japonica* throughout the six growth stages. The CGA content in a unit weight of tetraploid was higher than that of diploid *L. japonica* at all the growth stages (Fig. 1B). The tetraploid *L. japonica* showed the highest CGA content in unit weight at the S2 stage and the CGA content gradually decreased from S2 to S6 stages. The luteoloside content in a unit weight of diploid and tetraploid *L. japonica* displayed an almost a similar trend (Fig. 1C). The accumulation of luteoloside content of unit weight of both diploid and tetraploid *L. japonica* continuously increased from S1 to S3. Furthermore, tetraploid *L. japonica* indeed exhibited higher luteoloside content than that of diploid *L. japonica*. Interestingly, there was a huge fluctuation in the luteoloside contents from S4 to S6 stages, the luteoloside content of both diploid and tetraploid in unit weight displayed a significant decrease at S4. Then, we observed a sudden increase in the luteoloside contents of tetraploid *L. japonica* but not in diploid *L. japonica* at S5. While the luteoloside contents of tetraploid *L. japonica* showed a significant reduction, the diploid *L. japonica* exhibited a minor increase at the S6 stage. Interestingly, the luteoloside content of the unit weight of diploid *L. japonica* was higher than that of tetraploid *L. japonica* at S4 and S6 growth stages.

### Sample preparation, Illumina sequencing and de novo assembly

To compare the characterization of transcriptome between diploid and tetraploid *L. japonica*, we designed a paired-end sequencing strategy for the four stages (from the S3 to the S6 stages) of diploid and tetraploid *L. japonica*. RNA was extracted from the buds of diploid and tetraploid *L. japonica* from different stages (S3–S6). After cleaning and filtering the poor reads by using SOAP, a total of 590,59,615 clean reads with an average length of 100 bp were obtained (Tables S1 and S2). These reads were further assembled into 200,382 sequences (contigs) (Table 1). Our assembled data was comparatively better (N50 = 2,055 bp; Average length = 1,331 bp) than the previous transcriptome assembly of *L. japonica* by Rai et al. (2017), indicating a high quality of our transcriptome data.

**Table 1:** Summary and comparison of assembly statistics for de novo transcriptome assembly.

	No. of contigs	N50	Average length	*n* > 500	*n* > 1,000	Total length
Trinity^1^	200,382	2,055	1,331	153,975 (76.8%)	96,773 (48.3%)	266,774,118
Bridger^1^	260,647	1,662	899	121,935 (46.8%)	68,033 (26.1%)	234,294,476
CLC^2^	132,053	975	668	49,831 (37.7%)	22,776 (17.2%)	88,253,035
Trinity^2^	351,356	1,480	882	175,121 (49.8%)	97,341 (27.7%)	309,874,152
SOAPdenovo^2^	120,798	1,420	792	52,789 (43.7%)	29,066 (24.1%)	95,718,128

**Notes:**

1 This study.

2 Rai et al. (2017).

### Functional annotation and GO and KEGG characterization of *L. japonica* transcriptome assembly

The *L. japonica* unigenes were annotated by BLAST searches against several public databases. A statistical summary of these annotations is listed in Table S3. Among the 87,809 unigenes, 19,651 (22.38%) could be annotated in COG, 24,567 (27.98%) in GO, 33,566 (38.23%) in KEGG, 25,563 (29.11%) in KOG, 30,591 (34.84%) in Pfam, 36,612 (41.70%) in Swiss-Prot and 41,009 (46.70%) in NR databases. 45,633 (51.97%) unigenes could be assigned at least one putative function from one of these databases. There was remaining 48.03% of the unigenes had no significant protein matches in any databases. These non-matched unigenes might be novel or diverse proteins and long non-coding RNAs in *L. japonica*, or could be derived from less conserved 3′ or 5′ untranslated regions of the genes. GO functional annotations were used to classify gene functions; A total of 24,567 unigenes could be assigned to three domains (biological process, molecular function and cellular component) and 51 functional categories. Within the cellular component domain, the three most enriched categories were “cell” (11,731/47.75%), “cell part” (11,730/47.75%), and “organelle” (8,820/35.90%). In the molecular function domain, the three most matched categories were “catalytic activity” (13,151/53.53%), “binding” (12,565/51.15%), and “transporter activity” (1,643/6.69%). In the biological process domain, the three most common categories were “metabolic process” (17,611/71.69%), “cellular process” (14,952/60.86%), and “single-organism process” (12,345/50.25%). The most commonly assigned functional categories in each domain were almost consistent with the results from the previous studies of *L. japonica* (Rai et al., 2017).

**Figure 2:**
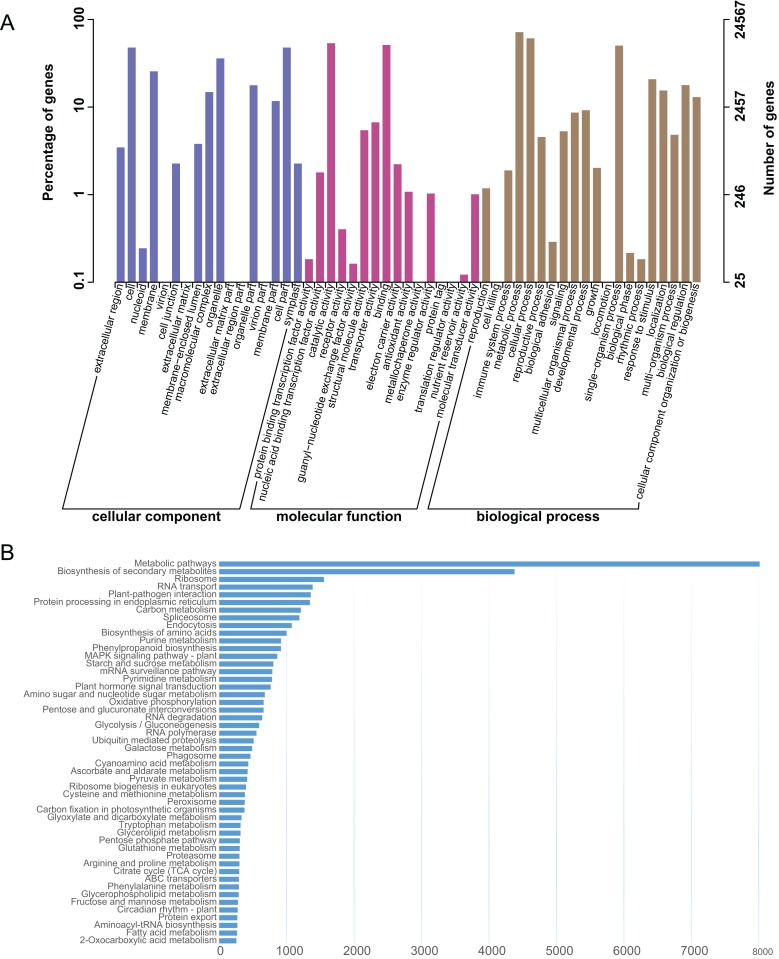
GO and KEGG annotation of the *L. japonica* unigenes. (A) GO annotation of all the *L. japonica* unigenes. Three primary GO categories and 51 subcategories (functional groups) are summarized into GO. (B) KEGG annotation of all the *L. japonica* unigenes. Only some representative and significant pathways are shown in the figure.

To systematically analyze different cellular components and their interactions within various metabolic pathways. An analysis of the KEGG database-based functional characterization of *L. japonica* was performed (Fig. 2A). In summary, 33,566 unigenes (38.23%) could be assigned to five main categories: metabolism; genetic information processing; environmental information processing; cellular process; and organismal systems with digestive system and environmental adaptation sub-categories (Fig. 2B). The most representative pathways were related to “Metabolic pathways” (8,013/23.87%), “Biosynthesis of secondary metabolites” (4,379/13.05%), and “Ribosome” (1,554/4.63%). Several key metabolic pathways, like phenylpropanoid biosynthesis, flavonoid biosynthesis and terpenoid backbone biosynthesis, which are involved in synthesizing precursors for the biosynthesis of CGA and secoiridoids, were assigned to 915, 135 and 164 unigenes, respectively. Identification and future characterization of transcripts involved in these different metabolic pathways will help us to better understand their functions in the biosynthesis of active compounds in *L. japonica*.

### Transcriptome expression analysis and differentially expressed genes between diploid and tetraploid *L. japonica*

**Figure 3:**
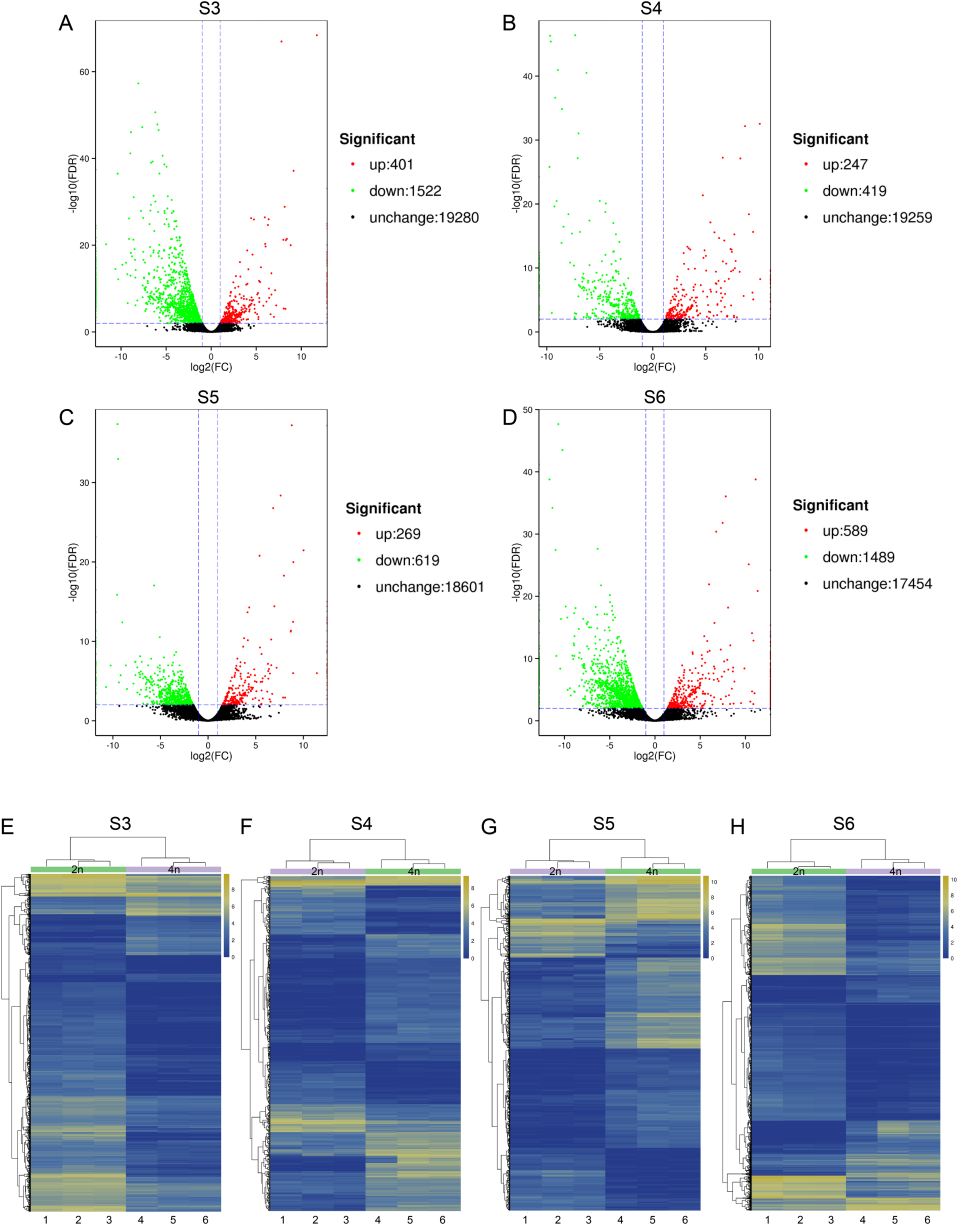
Differential gene expression analysis. (A–D) Volcano plots of the transcriptome between diploid and tetraploid *L. japonica* at four growth stages. (E–H) Heat map of the differentially expressed genes (DEGs) between diploid and tetraploid *L. japonica* at four growth stages.

**Figure 4:**
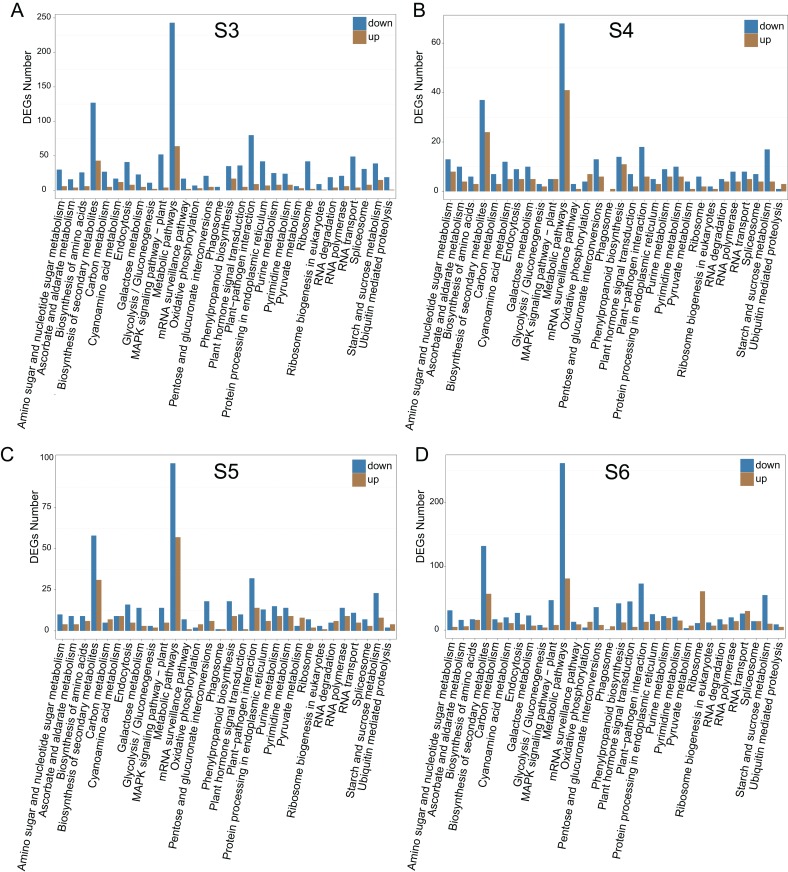
KEGG pathway enrichment of DEGs between diploid and tetraploid of *L. japonica* at four growth stages. KEGG pathway enrichment of DEGs between diploid and tetraploid of *L. japonica* at four growth stages. (A) S3 stage; (B) S4 stage; (C) S5 stage; and (D) S6 stage.

To relate the changes of phenotypes between diploid and tetraploid *L. japonica*, we calculated the expression abundance for unigenes between diploid and tetraploid *L. japonica* among various development stages. Here, clean paired-end reads were aligned to the de novo assembled transcriptome by using the Bowtie 2 program. Finally, the expression level of unigenes was determined as FPKM. The S4 stage of diploid and tetraploid *Lonicera japonica* showed the lowest number of transcriptionally active unigenes (FPKM > 0) (Table S4). Also, the diploid *L. japonica* exhibited higher transcriptional activity than the tetraploid *L. japonica* among all the development stages, especially the S3 and S5 stages of diploid *L. japonica*. The comparative transcriptomic study showed the S3 and S6 growth stages have 1,923 and 2,078 DEGs respectively (Figs. 3A–3H), which is two folds higher than that of S4 and S5 growth stages (666 and 888 DEGs respectively) (Table S5) (Figs. 3A–3H). Compared to the unigenes of diploid *L. japonica*, more transcripts displayed down-regulated expression rather than up-regulated expression during the growth process. For example, 401 transcripts showed up-regulated gene expression levels, but 1,522 displayed down-regulated gene expression levels at the S3 growth stage. Similarly, most of DEGs exhibited down-regulation rather than up-regulation at other growth stages. Also, a Venn diagram showed 163 unigenes showed changes among all S3–S6 growth stages (Fig. S2). Previous studies suggested the tetraploid *L. japonica* has excellent characteristics, such as higher accumulation of polyphenol components, higher yields of biomass as well as stronger resistance to heat stress than that of diploid *L. japonica*. To explore the possible genes regulating the secondary metabolism pathway, especially for CGA and luteoloside, we further performed KEGG enrichment analyses with the DEGs of tetraploid *L. japonica* at various growth stages (Figs. 4A–4D). Interestingly, the most DEGs present at all the growth stages were found to be mainly involved in the plant-pathogen interaction (Table 2). Additionally, genes participating in the Flavonoid and Anthocyanin biosynthesis also showed significant change during S3–S5 stages. Plant phenylpropanoids are one kind of anti-inflammatory agents. We observed the significant differential expression on genes involving in phenylpropanoid biosynthesis during all plant growth stages (Table 2). Besides the different gene expression on secondary metabolism, genes involved in plant hormone biosynthesis and signal transduction also showed remarkable changes between diploid and tetraploid *L. japonica* (Table 2), which might also be linked to their different phenotypes and secondary metabolite production.

**Table 2:** KEGG pathway enrichment of differential expression genes between diploid and tetraploid of *L. japonica*.

	2n vs 4n (S3)	2n vs 4n (S4)	2n vs 4n (S5)	2n vs 4n (S6)
KEGG pathway enrichment of DEG (*P* < 0.01)	Plant-pathogen interaction	Plant-pathogen interaction	Plant-pathogen interaction	Plant-pathogen interaction
	Cyanoamino acid metabolism	Tryptophan metabolism	Flavonoid biosynthesis	Phenylpropanoid biosynthesis
	Indole alkaloid biosynthesis	Phenylpropanoid biosynthesis	Galactose metabolism	RNA polymerase
	Plant hormone signal transduction	RNA polymerase	Phenylpropanoid biosynthesis	Starch and sucrose metabolism
	Flavonoid biosynthesis;	Galactose metabolism	Tryptophan metabolism	Galactose metabolism
	Starch and sucrose metabolism	ABC transporters	Zeatin biosynthesis	Glucosinolate biosynthesis
	Phenylpropanoid biosynthesis	Pyrimidine metabolism		
	Linoleic acid metabolism	Anthocyanin biosynthesis		

### Expression profile of unigenes involved in CGA and luteoloside biosynthesis pathways during the flower development

CGA and luteoloside biosynthesis is proposed to occur through three alternative routes as shown in Fig. S3. CGA biosynthesis and luteoloside biosynthesis share the same precursor (p-Coumaroyl-CoA). CGA biosynthesis is proposed to occur through three alternative routes where either p-coumaroyl quinate, p-coumaroyl shikimate, or caffeoyl-CoA could convert into CGA (Comino et al., 2007). While only p-Coumaroyl-CoA can be used for luteoloside biosynthesis (Niggeweg, Michael & Martin, 2004). To identify potential candidate unigenes from CGA and luteoloside biosynthetic pathways, we screened the assembled transcriptome of *L. japonica* and identified homologs for all the known enzymes involved in these pathways. To narrow down the most potential candidate unigenes associated with these key metabolic pathways as well as the accuracy of detected potential candidate unigenes, we further performed sequence filtering by combining the functional annotation and sequence similarity. Finally, this approach confirmed the identification of 84 unigenes associated with CGA and luteoloside biosynthesis pathways (Table S6).

We observed unigenes associated with the CGA and luteoloside biosynthesis between diploid and tetraploid *L. japonica* did not show a similar expression profile during the flower development. For the diploid *L. japonica*, C4H, 4CL, C3H and HQT were the four genes with remarkable changes (Fig. 4A). The CHS gene in diploid *L. japonica* displayed a continuous decreasing trend in its expression, with the major decrease from S3 to S4 and S5 to S6. In contrast, CHS of tetraploid *L. japonica* only showed a significant reduction from S3 to S4, but no significant reduction was observed during S4–S6. The expression profile of the 4CL exhibited a similar trend between diploid and tetraploid *L. japonica*. The only difference is that the expression level of the 4CL gene of diploid still kept increasing at S5–S6 stage, while a reduced trend of 4CL expression was observed in tetraploid *L. japonica* (Fig. S4). The expression value of FSII displayed a significant decrease at the S5 to the S6 development stage of tetraploid *L. japonica*, while the expression profile of FSII was almost stable at different stages in diploid *L. japonica*. The expression value of the HQT gene of both diploid and tetraploid *L. japonica* displayed remarkably increasing with the growth of *L. japonica*. Interestingly, the increased expression of HQT of diploid *L. japonica* was mainly at the S5–S6 stage, while the enhanced expression level of HQT in tetraploid *L. japonica* was mainly at the S4–S5 stage. In contrast with the expression profile of FSII, the C3H and CHI genes also showed significant expression change in diploid, but the most stable expression was noticed among tetraploid *L. japonica* (Fig. S4).

### Comparing the expression of unigenes involved in secoiridoid biosynthesis pathways between diploid and tetraploid *L. japonica* at various development stages

**Figure 5:**
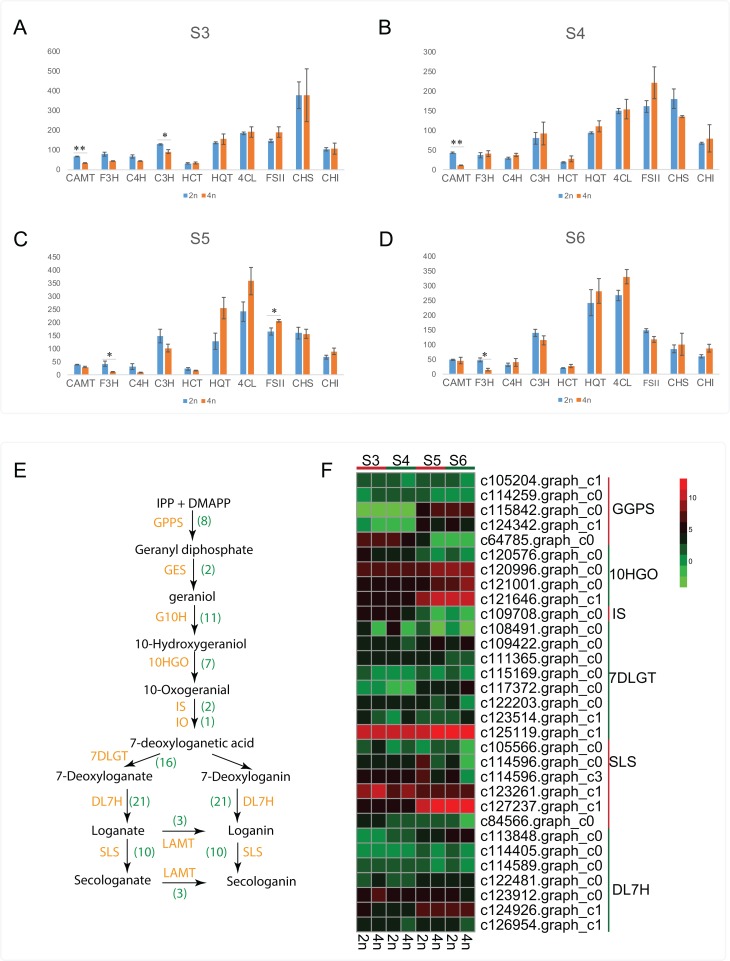
Cluster analysis of DEGs related to CGA, luteolosides and secoiridoid biosynthesis between diploid and tetraploid *L. japonica* at various developmental stages. (A–D) Transcript expression analysis for unigenes associated with CGA, and luteolin biosynthetic pathways. Error bars are ± SE, *n* ≥ 3. (E and F) Proposed secoiridoid metabolic pathways in *L. japonica*. Numbers in the bracket denote the identified gene copy number and transcript expression analysis for unigenes associated with secoiridoid biosynthetic pathways is shown in F.

Apart from the CGA and luteoloside, secoiridoid such as loganin, secologanin, sweroside and kingiside among others have been identified from the extracts of *L. japonica* that might synergistically work with CGA, luteoloside and other effective components of *L. japonica* to play a pharmacodynamics (Ghisalberti, 1998; Xiong et al., 2014). Previous studies also suggested secoiridoids featured with pharmaceutically active and were known to possess the effects of anti-tumor, anti-inflammatory and antioxidant activities (Ghisalberti, 1998; Suksamrarn et al., 2002; Viljoen, Mncwangi & Veermak, 2012; Rai et al., 2017). By a stringent screening of our transcriptome assembly, we identified 81 unigenes representing all known enzymes from secoiridoid metabolic pathways (Table S6). Transcript expression analysis for unigenes associated with secoiridoid biosynthesis pathways between diploid and tetraploid *L. japonica* at various development stages are shown in Figs. 5E and 5F. About 31 genes corresponding to secoiridoid biosynthesis pathways exhibited significant differences between diploid (2n) and tetraploid (4n). The most significant change of gene expression level appeared at S5 stages, where 18 genes displayed significant changes in their expression level (15 genes showed remarkable change at S6, 9 genes at S3, and 8 genes at S4 stage). This is similar to the expression of CGA and luteoloside biosynthesis, where many genes showed the most significant changes at the S5 growth stage.

### A comprehensive view of the gene expression profile of the main metabolic pathways

**Figure 6:**
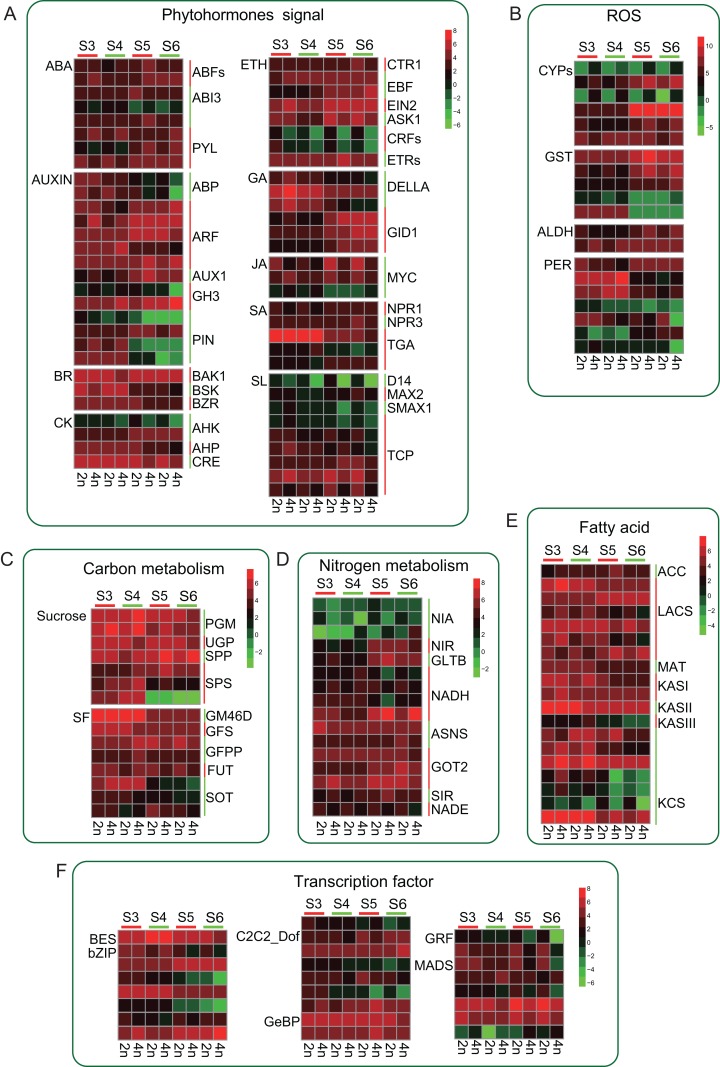
Schematic summary of the effects of gene expression profile of metabolic flux of CGA and luteolosides biosynthesis pathway. (A–F) Small arrows indicate increase (upwards) or decrease (downwards); green color denotes the diploid and red color denotes the tetraploid. The big arrows denote the changes in metabolite flux of CGA and luteolosides biosynthesis pathway.

Tetraploid *L. japonica* generally features bigger flowers or buds compared to that of diploid (Fig. 1A). We, therefore, analyzed the genes of the phytohormones signaling pathway and many TFs that might participate in the regulation of flower development. We found several TFs involving in the flower development showed significant changes (Fig. 6). However, there were no significant changes between diploid and tetraploid. For instance, MADS genes play a key role in plant body formation and flower growth (Egea-Cortines, Saedler & Sommer, 1999; Bielenberg et al., 2008). We found that, while few MADS gene copies showed higher expression levels in 2n, some copies displayed increased gene expression levels in 4n, and even some genes showed varying expression levels at different stages of flower development, which might be associated with their specific functions. Likewise, Auxin is also involved in regulating flower development (Okushima et al., 2005). A similar trend was observed for this gene as well; there was no consistent change in gene expression level. Furthermore, we observed that many genes mainly changed at S5 and S6 stages. Many PIN genes and ABP genes of tetraploid *L. japonica* displayed reduced gene expression levels than that of diploid at S5 and S6 stages. However, GH3, AUX1 and ARFs from the tetraploid *L. japonica* showed up-regulation than that of diploid. Not only the TFs, but tetraploid *L. japonica* also have differential gene expression level in another crucial metabolism such as phytohormones signaling pathway, anti-oxidant stress, fatty acid and nitrogen metabolism. Thus, our results displayed a comprehensive view of the effects of the changed ploidy level of *L. japonica* (2n vs 4n) on vital gene expression profiles for the key metabolic pathways.

### Identification of single-nucleotide polymorphisms and simple sequence repeats

SNP is a variation at a single nucleotide position in DNA sequence among individuals of the same species and they are the most common DNA polymorphisms in genome sequences of all the species (Baird et al., 2008; Wagner et al., 2013). They are thought to play a major role in the induction of phenotypic variations. SNP is usually treated as available molecular markers to use for analysis of genomic variations in plants, their association mapping as well as diagnostics, evolutionary studies analysis, fingerprinting and also widely molecular breeding applications (Filliol et al., 2006). In this study, the SNP sites were identified by using the GATK SNP calling the process of RNA-seq. Finally, we obtained the SNP number of diploid and tetraploid *L. japonica* at different growth stages (Table S7). SSRs are the tandem iterations of short oligonucleotides ubiquitously distributed within the genome and it also can serve as an important marker for determining genetic variations, including paternity determination, population genetics studies, genetic diversity assessment and for the development of genetic maps. To identify SSRs for *L. japonica*, we searched transcriptome assembly for mono- to hexanucleotide motifs with a minimum of ten repetitions using MISA software. Overall, we identified 16,554 SSRs across 26,173 unigenes, with 1,016 unigenes having more than one SSR (Table S7). Within the identified SSRs, mono-nucleotide represented the largest fraction (7,915/47.18%) of all SSRs, followed by and di-nucleotides (5,133/31.01%) and tri-nucleotides (2,206/13.33%). The number of SSRs identified as tetra-, penta- and hexanucleotide repeat classes were relatively small. Identified SNPs and SSRs of *L. japonica* in this study may provide potential genetic markers, which will be important for population genetics, comparative genomic studies and molecular breeding application across different species or eco-types.

## Discussion

*Lonicera japonica* is an effective traditional Chinese medicine for thousands of years being used as an anti-inflammatory and anti-nociceptive (Shang et al., 2011). Previous studies have revealed that the pharmacological property of *L. japonica* might be attributed to its bioactive components, including phenolic acids and flavonoids (Cai et al., 2004; Li, Kong & Wu, 2018). Additionally, the potential molecular mechanisms that produce the bioactive biosynthesis of CGA and luteoloside are still not comprehensively understood in *L. japonica* (Chen et al., 2015; Rai et al., 2017). The majority of previous studies indicated that chromosome doubling causes significant changes in morphology and physiology but may also increase the content of secondary metabolites, particularly in medicinal plants, enhance growth rates and improve the genetic quality. Our previous study also demonstrated this result that the tetraploid *L. japonica* showed higher biomass yields and polyphenol contents and stronger antioxidant activity than those in their diploid plants (Kong et al., 2017a).

However, the molecular mechanism underlying CGA and luteoloside biosynthesis between diploid and tetraploid *L. japonica* remains unclear. Therefore, to investigate the underlying mechanisms between diploid and tetraploid *L. japonica*, we used Illumina HiSeq™ 4,000 platform to understand gene expression profiles and the key pathways for CGA, luteoloside and secoiridoid biosynthesis in *L. japonica*. We obtained an excellent quality of transcriptome assembly with longer contig N50 compared to the previously published *L. japonica* transcriptome. Finally, a total of 87,809 unigenes were obtained and RNA-Seq analysis was performed on DEGs between diploid and tetraploid *L. japonica* at different growth stages based on these data.

Apart from the higher yield of bioactive components in autotetraploid *L. japonica*, previous studies have also shown its stronger disease resistance compared to the diploid plant (Kong et al., 2017a, 2017b; Li, Kong & Wu, 2018). Our analysis of KEGG pathway enrichment of DEGs between diploid and tetraploid of *L. japonica* at four different flowering growth stages showed the increased gene expression level of genes involving in the plant-pathogen interaction, reflecting that tetraploid *L. japonica* has better pathogen-resistance than the diploid one. Besides, DEGs between diploid and tetraploid of *L. japonica* were also enriched in another important metabolism such as plant hormone signal transduction, carbon metabolism and starch/sucrose metabolism. These metabolisms are closely associated with the plant growth and development process. Therefore, the excellent characteristics of tetraploid *L. japonica* might have resulted from the DEGs significant changes in these metabolic pathways. Corresponding to the higher phenolic acids and flavonoids in tetraploid of *L. japonica*, many DEGs were also enriched in the metabolism of Flavonoid biosynthesis and Phenylpropanoid biosynthesis and most of these genes displayed increased gene expression level compared to diploid.

**Figure 7:**
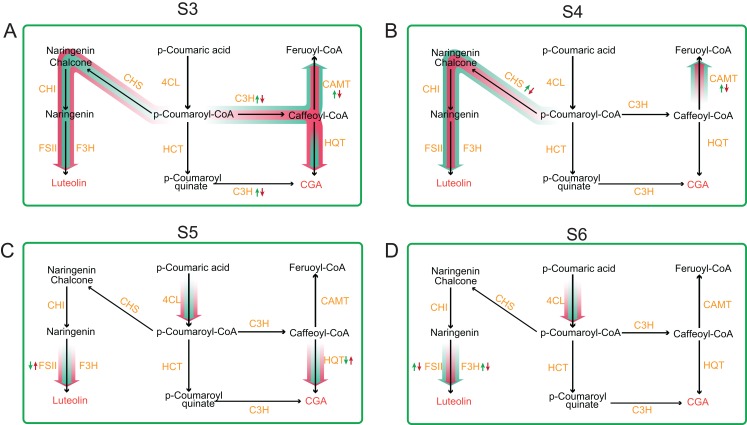
Comprehensive view of the gene expression profile of main metabolic pathways between diploid and tetraploid *L. japonica* at four different growth stages. (A) S3 stage; (B) S4 stage; (C) S5 stage; and (D) S6 stage.

Our result of CGA and luteoloside contents in unit weight tetraploid *L. japonica* showed a significant increase in comparison to those of diploid *L. japonica*, which almost keep a similar trend of our previous results of CGA and luteoloside contents based on 100 flowers of tetraploid *L. japonica*. Next, we analyzed the genes expression profile of CGA, luteoloside and secoiridoid biosynthesis pathways at different growth. Only CAMT and C3H showed significant changes of expression level, relating with the phenotypic results, hence we hypothesized that the increased CGA and luteoloside contents of tetraploid *L. japonica* might have resulted from the lower expression level of CAMT and C3H, leading to the lower conversion of Caffeoyl-CoA to Feruoyl-CoA and higher accumulation of the CGA and luteolin at S3 growth stage (Fig. 7). We observed that luteoloside content in unit weight among tetraploid *L. japonica* was lower than that of diploid *L. japonica* at the S4 growth stage. This was further corroborated by our transcriptome analysis, where the gene expression level of the CHS gene was significantly decreased and was lower than that of diploid *L. japonica*. Besides the CHS, the CAMT was another gene that also showed significant changes in expression level at the S4 growth stage. Similar to the S3 growth stage, the higher yields of CGA might be caused by the reduced usage of the precursor of CGA, Caffeoyl-CoA, in tetraploid *L. japonica*. Alternatively, we also observed an enormously decreased yields of luteoloside both in diploid and tetraploid *L. japonica* at S3 and S4. With no surprise, following the phenotypic observation, the expression level of CHS had a massive reduction from S3 to S4 growth stage in both diploid and tetraploid *L. japonica*, which might explain why there was a sudden decrease in the accumulation luteoloside during these stages. Besides, the expression level of HQT, 4CL and FSII of tetraploid *L. japonica* were higher than those of diploid. By associating the CGA and luteoloside content between diploid and tetraploid *L. japonica*, we indeed found some biggest differences between CGA and luteoloside yield at the S5 growth stage. Additionally, we also observed a significantly decreased expression level of F3H, which might suggest that the higher accumulation of luteoloside of tetraploid *L. japonica* at S5 growth stage was caused by enhanced gene expression of FSII, not due to the decreased expression of F3H gene. However, at the S6 stage, we observed both F3H and FSII of tetraploid *L. japonica* showed remarkably lower expression levels than those of diploid, which might result in the lower luteoloside content in a unit weight of tetraploid *L. japonica* than that of diploid. Thus, these results highlighted the dynamic changes among different metabolic pathways between diploid and tetraploid *L. japonica*. Overall, our work provides a foundation for further studying these important secondary metabolite pathways and their implications in traditional Chinese/Japanese medicine.

## Conclusion

In summary, we sequenced the transcriptome of diploid and tetraploid *L. japonica* and provided the best transcriptome assembly of *L. japonica* thus far. We, therefore, investigated the metabolic difference between diploid and tetraploid *L. japonica* through the DEGs at different growth stages. The KEGG enrichment of DEGs displayed that tetraploid *L. japonica* many genes involved in plant-pathogen interaction, phenylpropanoid biosynthesis, flavonoid biosynthesis and plant hormone signal transduction etc. which might endow more excellent characteristics of tetraploid *L. japonica* than diploid, such as high yield, excellent quality, strong resistance. By associating with the phenotypic data and gene expression profile of CGA and luteoloside and related transcription factor as well as genes participating in the phytohormone signaling pathway between diploid and tetraploid *L. japonica* at different growth stages, our results characterize a potential molecular regulatory mechanism for CGA and luteoloside biosynthesis of tetraploid *L. japonica* at different growth stages. However, further experimental validation of these identified genes, metabolites and their subsequent regulations in future studies will enable us to gain more detailed insights.

## Supplemental Information

10.7717/peerj.8690/supp-1 Supplemental Information 1 Supplemental Tables.

10.7717/peerj.8690/supp-2 Supplemental Information 2 Supplemental Figures.

10.7717/peerj.8690/supp-3 Supplemental Information 3 Raw data for chlorogenic acid content.

## Abbreviations

GPPS: Geranyl diphosphate synthase
GES: Geraniol synthase
G10H: Geraniol 10-hydroxylase
10HGO: 8-Hydroxygeraniol oxidoreductase
IS: Iridoid synthase
IO: Iridoid oxidase
7DLGT: 7-Deoxyloganetic acid glucosyl transferase
DL7H: Deoxyloganin 7-hydroxylase
LAMT: Loganic acid *O*-methyltransferase
SLS: Secologanin synthase
PAL: Phenylalanine ammonia lyase
C4H: Cinnamic acid 4-hydroxylase
C3H: p-Coumaric acid 3-hydroxylase
4CL: 4-Hydroxycinnamoyl-CoA ligase/4coumarate-CoA ligase
HCT: Hydroxycinnamoyl-CoA shikimate/quinate hydroxycinnamoyl transferase
HQT: Hydroxycinnamoyl-CoA quinate hydroxycinnamoyl transferase
CHS: Chalcone synthase
CHI: Chalcone isomerase
F3H: Flavonoid 3-monooxgenase
FSII: Flavonol synthase
CCoAMT: Caffeoyl-CoA-*O*-methyltransferase
CGA: Chlorogenic acid
ROS: Reactive oxygen species
ABA: Abscisic acid
BRs: Brassinosteroids
CK: Cytokinins
ETH: Ethylene
GAs: Gibberellins
JA: Jasmonic acid
SL: Strigolactones
SF: Sulfate Fucan
BES: BRI1-EMS suppressor
GeBP: GL1 enhancer binding protein
GRF1: Growth-Regulating factor1
MADS: MADS-box transcription factor
CYP: Cytochrome P450
GST: Glutathione S-transferase
ALDH: Aldehyde dehydrogenase
PER: peroxidase
GH3: Glycoside Hydrolase 3
AUX1: Auxin transporter protein 1
ARF: Auxin response factor

## Additional Information and Declarations

### Data Availability

The following information was supplied regarding data availability: The transcriptome assemblies for *L. japonica* in this study are available at the China National Gene Bank under the accession number CNA0003265. These data are also available in the CNGB Nucleotide Sequence Archive (CNSA: http://db.cngb.org/cnsa; accession number CNS0095592–CNS0095615). Additional data containing the links between the annotations (using the GO: terms), and the sequence data can be assessed at: Wang, Hongli (2020): L.japonica.Unigene.anno. figshare. Dataset. DOI 10.6084/m9.figshare.11618199.v1.

## References

[ref-1] Baird NA, Etter PD, Atwood TS, Currey MC, Shiver AL, Lewis ZA, Selker EU, Cresko WA, Johnson EA (2008). Rapid SNP discovery and genetic mapping using sequenced RAD markers. PLOS ONE.

[ref-2] Bielenberg DG, Wang Y(Eileen), Li Z, Zhebentyayeva T, Fan S, Reighard GL, Scorza R, Abbott AG (2008). Sequencing and annotation of the evergrowing locus in peach [*Prunus persica* (L.) Batsch] reveals a cluster of six MADS-box transcription factors as candidate genes for regulation of terminal bud formation. Tree Genetics & Genomes.

[ref-3] Cai Y, Luo Q, Sun M, Corke H (2004). Antioxidant activity and phenolic compounds of 112 traditional Chinese medicinal plants associated with anticancer. Life Sciences.

[ref-4] Chen C-Y, Peng W-H, Tsai K-D, Hsu S-L (2007). Luteolin suppresses inflammation-associated gene expression by blocking NF-κB and AP-1 activation pathway in mouse alveolar macrophages. Life Sciences.

[ref-5] Chen Z, Tang N, You Y, Lan J, Liu Y, Li Z (2015). Transcriptome analysis reveals the mechanism underlying the production of a high quantity of chlorogenic acid in young leaves of lonicera macranthoides Hand.-Mazz. PLOS ONE.

[ref-6] Comai L (2005). The advantages and disadvantages of being polyploid. Nature Reviews Genetics.

[ref-7] Comino C, Portis E, Acquadro A, Pinelli P, Hehn A, Bourgaud F, Lanteri S (2007). Isolation of a hydroxycinnamoyltransferase involved in phenyl-propanoid biosynthesis in *Cynara cardunculus* L. Acta Horticulturae.

[ref-8] De Weathers L, Jesus-Gonzalez PJ (2003). Tetraploid Artemisia annua hairy roots produce more artemisinin than diploids. Plant Cell Reports.

[ref-9] Dehghan E, Häkkinen ST, Oksman-Caldentey K-M, Ahmadi FS (2012). Production of tropane alkaloids in diploid and tetraploid plants and in vitro hairy root cultures of Egyptian henbane (*Hyoscyamus muticus* L.). Plant Cell, Tissue and Organ Culture.

[ref-10] Egea-Cortines M, Saedler H, Sommer H (1999). Ternary complex formation between the MADS-box proteins SQUAMOSA, DEFICIENS and GLOBOSA is involved in the control of floral architecture in *Antirrhinum majus*. EMBO Journal.

[ref-11] Filliol I, Motiwala AS, Cavatore M, Qi W, Hazbón MH, Del Valle MB, Fyfe J, García-García L, Rastogi N, Sola C, Zozio T, Guerrero MI, León CI, Crabtree J, Angiuoli S, Eisenach KD, Durmaz R, Joloba ML, Rendón A, Sifuentes-Osornio J, Ponce De Leon A, Cave MD, Fleischmann R, Whittam TS, Alland D (2006). Global phylogeny of Mycobacterium tuberculosis based on single nucleotide polymorphism (SNP) analysis: Insights into tuberculosis evolution, phylogenetic accuracy of other DNA fingerprinting systems, and recommendations for a minimal standard SNP set. Journal of Bacteriology.

[ref-12] Ghisalberti EL (1998). Biological and pharmacological activity of naturally occurring iridoids and secoiridoids. Phytomedicine.

[ref-13] Huyut Z, Beydemir Ş, Gülçin I (2017). Antioxidant and antiradical properties of selected flavonoids and phenolic compounds. Biochemistry Research International.

[ref-14] Kong D, Li Y, Bai M, Deng Y, Liang G, Wu H (2017a). A comparative study of the dynamic accumulation of polyphenol components and the changes in their antioxidant activities in diploid and tetraploid *Lonicera japonica*. Plant Physiology and Biochemistry.

[ref-15] Kong D-X, Li Y-Q, Bai M, He H-J, Liang G-X, Wu H (2017b). Correlation between the dynamic accumulation of the main effective components and their associated regulatory enzyme activities at different growth stages in *Lonicera japonica* Thunb. Industrial Crops and Products.

[ref-16] Lee EJ, Kim JS, Kim HP, Lee J-H, Kang SS (2010). Phenolic constituents from the flower buds of *Lonicera japonica* and their 5-lipoxygenase inhibitory activities. Food Chemistry.

[ref-17] Li Y, Kong D, Wu H (2018). Comprehensive chemical analysis of the flower buds of five *Lonicera* species by ATR-FTIR, HPLC-DAD, and chemometric methods. Revista Brasileira de Farmacognosia.

[ref-18] Niggeweg R, Michael AJ, Martin C (2004). Engineering plants with increased levels of the antioxidant chlorogenic acid. Nature Biotechnology.

[ref-19] Okushima Y, Mitina I, Quach HL, Theologis A (2005). Auxin Response Factor 2 (ARF2): a pleiotropic developmental regulator. Plant Journal.

[ref-20] Rai A, Kamochi H, Suzuki H, Nakamura M, Takahashi H, Hatada T, Saito K, Yamazaki M (2017). De novo transcriptome assembly and characterization of nine tissues of *Lonicera japonica* to identify potential candidate genes involved in chlorogenic acid, luteolosides, and secoiridoid biosynthesis pathways. Journal of Natural Medicines.

[ref-21] Repčák M, Krausová T (2009). Phenolic glucosides in the course of ligulate flower development in diploid and tetraploid Matricaria chamomilla. Food Chemistry.

[ref-22] Shang X, Pan H, Li M, Miao X, Ding H (2011). *Lonicera japonica* Thunb.: ethnopharmacology, phytochemistry and pharmacology of an important traditional Chinese medicine. Journal of Ethnopharmacology.

[ref-23] Simbolo M, Gottardi M, Corbo V, Fassan M, Mafficini A, Malpeli G, Lawlor RT, Scarpa A (2013). DNA qualification workflow for next generation sequencing of histopathological samples. PLOS ONE.

[ref-24] Soltis PS, Marchant DB, Van De Peer Y, Soltis DE (2015). Polyploidy and genome evolution in plants. Current Opinion in Genetics & Development.

[ref-25] Suksamrarn A, Kumpun S, Kirtikara K, Yingyongnarongkul B, Suksamrarn S (2002). Iridoids with anti-inflammatory activity from *Vitex peduncularis*. Planta Medica.

[ref-26] Viljoen A, Mncwangi N, Veermak I (2012). Anti-Inflammatory iridoids of botanical origin. Current Medicinal Chemistry.

[ref-27] Wagner CE, Keller I, Wittwer S, Selz OM, Mwaiko S, Greuter L, Sivasundar A, Seehausen O (2013). Genome-wide RAD sequence data provide unprecedented resolution of species boundaries and relationships in the Lake Victoria cichlid adaptive radiation. Molecular Ecology.

[ref-28] Wang Z, Clifford MN, Sharp P (2008). Analysis of chlorogenic acids in beverages prepared from Chinese health foods and investigation, in vitro, of effects on glucose absorption in cultured Caco-2 cells. Food Chemistry.

[ref-29] Wang T, Yang B, Guan Q, Chen X, Zhong Z, Huang W, Zhu W (2019). Transcriptional regulation of *Lonicera japonica* Thunb. during flower development as revealed by comprehensive analysis of transcription factors. BMC Plant Biology.

[ref-30] Xiong Y, Xiao X, Yang X, Yan D, Zhang C, Zou H, Lin H, Peng L, Xiao X, Yan Y (2014). Quality control of *Lonicera japonica* stored for different months by electronic nose. Journal of Pharmaceutical and Biomedical Analysis.

[ref-31] Yuan Y, Wang Z, Jiang C, Wang X, Huang L (2014). Exploiting genes and functional diversity of chlorogenic acid and luteolin biosyntheses in *Lonicera japonica* and their substitutes. Gene.

