# Supplementary material for: Comparative transcriptomic analyses of chlorogenic acid and luteolosides biosynthesis pathways at different flowering stages of diploid and tetraploid Lonicera japonica
Source: PeerJ. 2020 Mar 6;8:e8690. doi: 10.7717/peerj.8690 (PMC7061910; doi:10.7717/peerj.8690)
Supplement: Supplemental Information 2 [file peerj-08-8690-s002.pdf]

Supplementary Material

1 Supplementary Figure

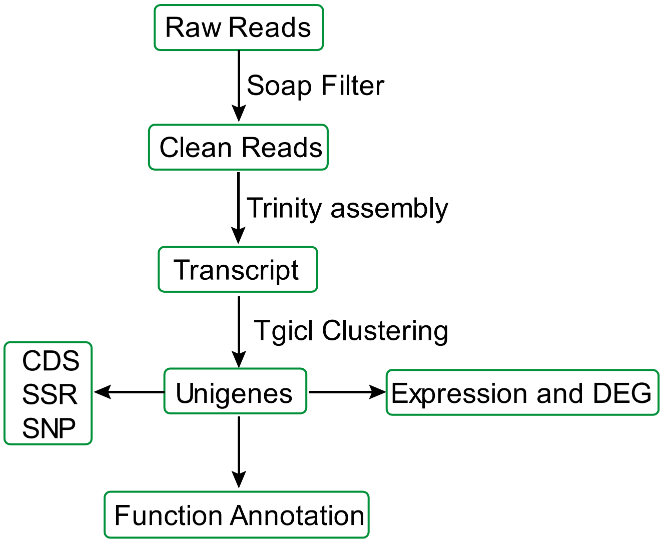

Supplementary Figure 1. Transcriptome analysis of *Lonicera japonica*

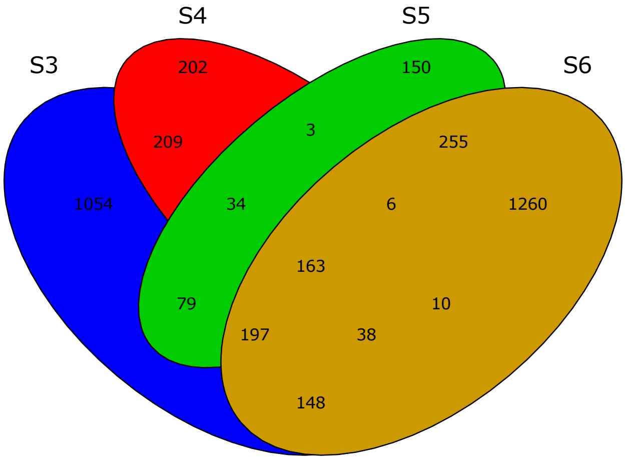

Supplementary Figure 2. A venn diagram showing differentially expressed unigenes among all S3-S6 growth stages of diploid and tetraploid *L. japonica*.

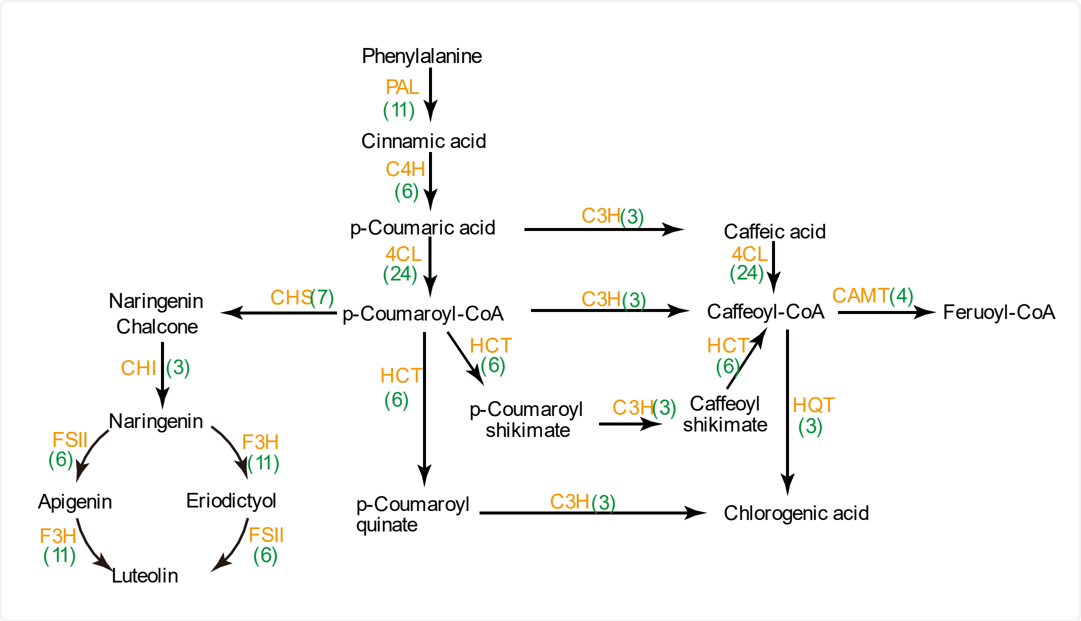

Supplementary Figure 3. Proposed CGA, and luteolin biosynthetic pathways in L. japonica. Numbers in the bracket is the identified gene copy number.

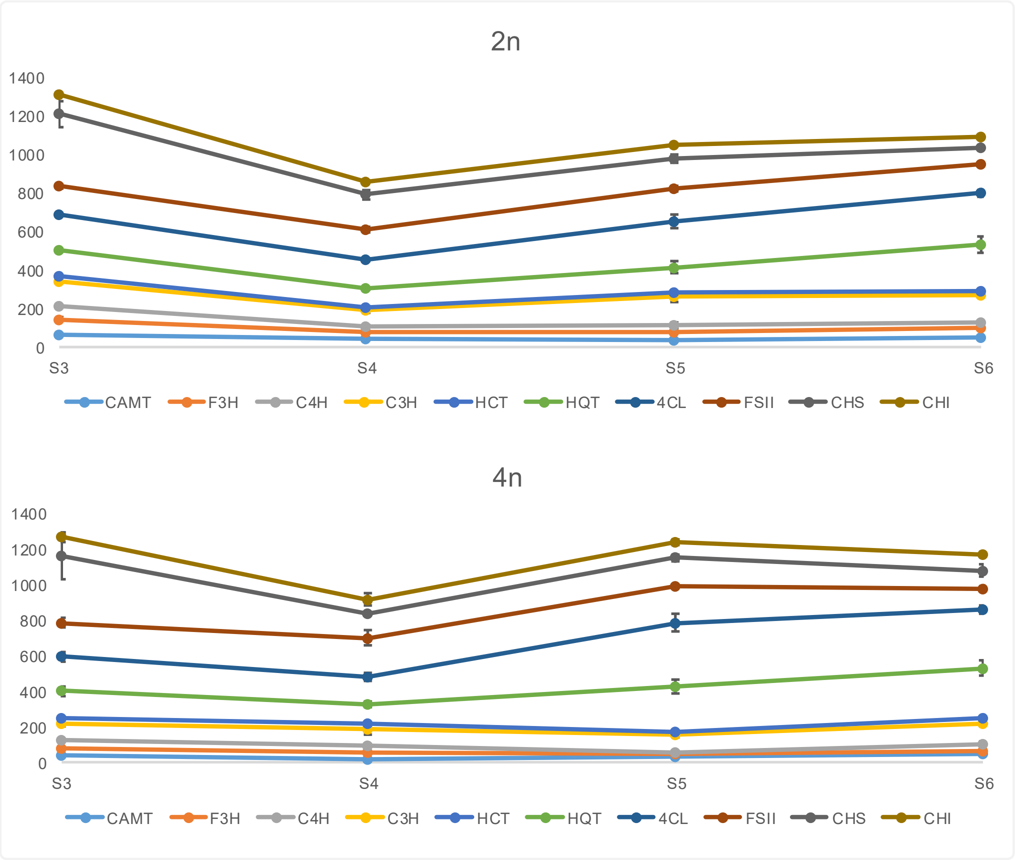

Supplementary Figure 4. Expression profile of unigenes involved in CGA and luteoloside biosynthesis pathways along the development of diploid and tetraploid *L. japonica*.
